# Supplementary figures and images for: Genomic Analysis of Response to Neoadjuvant Chemotherapy in Esophageal Adenocarcinoma
Source: Cancers (Basel). 2021 Jul 6;13(14):3394. doi: 10.3390/cancers13143394 (PMC8308111; doi:10.3390/cancers13143394)

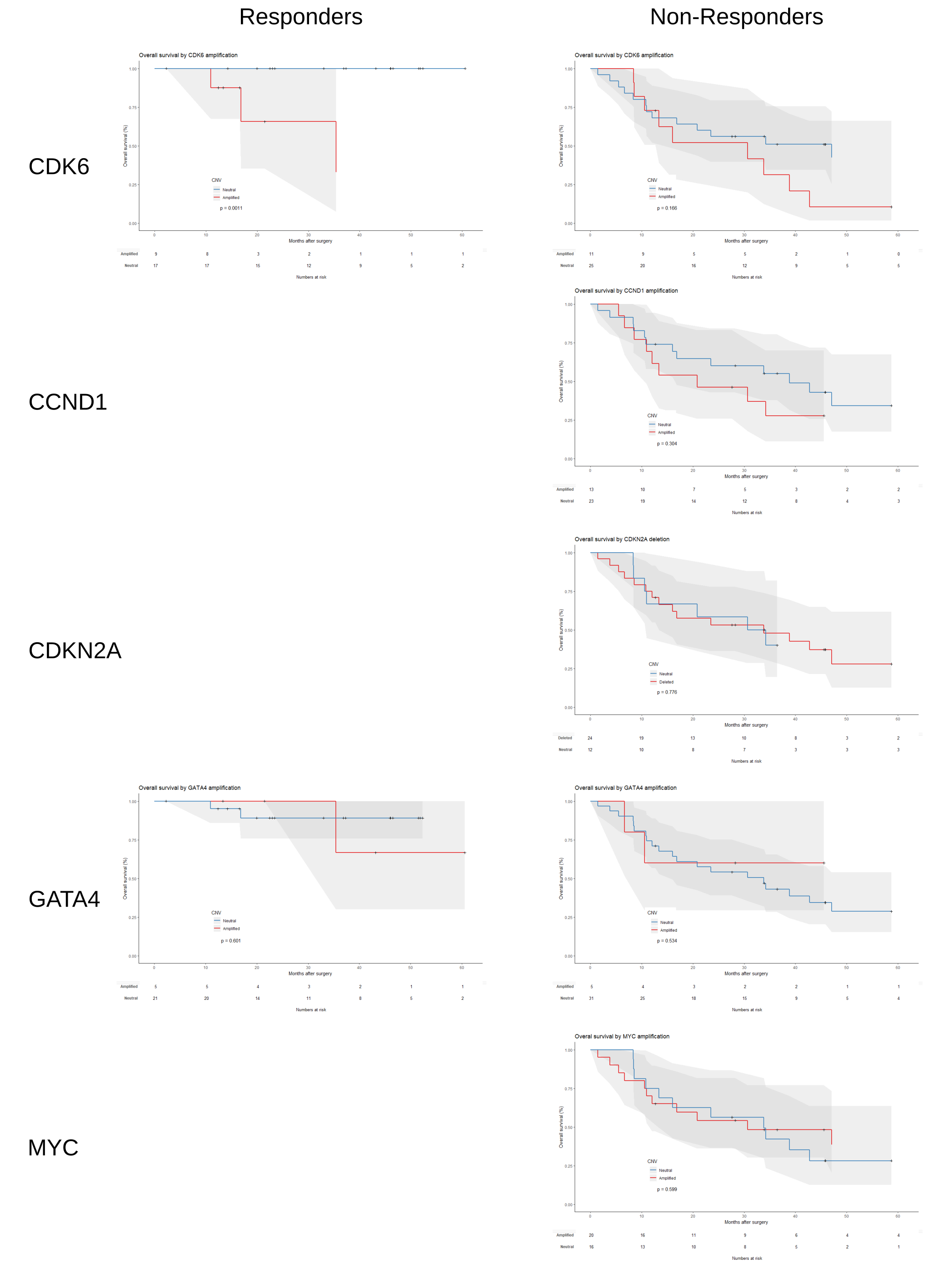

Supplement: Supplementary file 1 [file cancers-13-03394-s001.zip › Supplementary Files/Supplementary Figure S1.png]
